# The role of microrna-346 in prostate cancer progression: Clinical significance and biomarker potential

**DOI:** 10.5937/jomb0-58279

**Published:** 2025-11-05

**Authors:** Lei Wang, Yanqing Wang, Li Tian, Zhengwei Wang, Xiaoqiang Liu, Guangzhou Cheng

**Affiliations:** 1 Tianjin Medical University General Hospital, Department of Urology, Tianjin, 300052, China; 2 Tengzhou Central People's Hospital, Department of Urology, Tengzhou, 277500, China

**Keywords:** prostate cancer, benign prostatic hyperplasia, hormone-sensitive prostate cancer, castration-resistant prostate cancer, miR-346, rak prostate, benigna hiperplazija prostate, hormonski osetljiv rak prostate, rak prostate rezistentan na kastraciju, miR-346

## Abstract

**Background:**

This study aimed to analyse the expression changes and clinical significance of microRNA-346 (miR-346) in PCa genesis, development, and hormone resistance transition.

**Methods:**

Data of the middle-aged and elderly male patients who were treated in Tengzhou Central People's Hospital from January 2017 to December 2023 were collected. The tissues and corresponding preoperative serum of 128 patients with newly diagnosed PCa, 130 patients with benign prostatic hyperplasia (BPH), 120 patients with hormone-sensitive PCa (HSPC), 116 patients with castration-resistant PCa (CRPC), and 150 healthy males of the same age who underwent physical examination in the physical examination centre of our hospital were collected. A quantitative real-time polymerase chain reaction was used to detect the relative expression of miR-346 in the subjects' tissues and serum. GraphPad Prism statistical analysis software was utilised to analyse the expression changes of miR-346 in the occurrence and progression of PCa and its correlation with clinical and pathological features.

**Results:**

The expression of miR-346 was significantly lower in PCa patients than in BPH patients and healthy population (P &lt; 0.001), while there was no difference between BPH and healthy population (P= 0.516). Tissue and serum expression were positively correlated (P&lt; 0.001). miR-346 was negatively correlated with tPSA, PHI, clinical stage, and Gleason score in PCa tissue/serum (P&lt; 0.001). Serum miR-346 in CRPC patients was negatively correlated with time to transformation (r= 0 .6 5 7 5 ) and survival (r= 0.5699) (P&lt; 0.001). Serum miR-346 was positively correlated with tPSA and ALP in HSPC and CRPC patients (P&lt; 0.001).

**Conclusions:**

Serum miR-346 expression in hormone resistance transition in PCa is gradually increasing, making it a potential biomarker for monitoring PCa progression.

## Abstract (sr)

**Uvod:**

Cilj ; je bio da se analizira promena ekspresije i klinički značaj mikroRNA-346 (miR-346) u nastanku, razvoju i prelasku raka prostate (PCa) u hormonski rezistentni oblik.

**Metode:**

Prikupljeni su podaci sredovečnih i starijih muškaraca koji su se lečili u Centralnoj narodnoj bolnici Tengdžou u periodu od januara 2017. do decembra 2023. godine. Prikupljeni su uzorci tkiva i odgovarajućeg preoperativnog seruma 128 pacijenata sa novodijagnostikovanim rakom prostate, 130 pacijenata sa benignom hiperplazijom prostate (BHP), 120 pacijenata sa hormonski osetljivim rakom prostate (HSPC), 116 pacijenata sa kastraciono rezistentnim rakom prostate (CRPC), kao i 150 zdravih muškaraca istih godina koji su obavili sistematski pregled u našem dijagnostičkom centru. Kvantitativna reverzna transkriptaza polimerazna lančana reakcija u realnom vremenu (qRT-PCR) je korišćena za detekciju relativne ekspresije miR-346 u tkivima i serumu ispitanika. Za analizu promena ekspresije miR-346 u nastanku i progresiji raka prostate i njene korelacije sa kliničkim i patološkim karakteristikama korišćen je statistički softver GraphPad Prism.

**Rezultati:**

Ekspresija miR-346 je bila značajno niža kod pacijenata sa rakom prostate u poređenju sa pacijentima sa BHP i zdravom populacijom (P&lt; 0,001), dok nije bilo razlike između BHP i zdrave populacije (P= 0,516). Ekspresija u tkivu i serumu bila je pozitivno korelisana (P&lt; 0,001). miR-346 je negativno korelisan sa ukupnim PSA (tPSA), PHI, kliničkom fazom i Glisonovim skorom u tkivu/serumu pacijenata sa rakom prostate (P&lt; 0,001). Serumski miR-346 kod CRPC pacijenata bio je negativno korelisan sa vremenom do transformacije (r= 0,6575) i preživljavanjem (r= 0,5699) (P&lt; 0,001). Serumski miR-346 bio je pozitivno korelisan sa tPSA i ALP kod pacijenata sa HSPC i CRPC (P&lt; 0,001).

**Zaključak:**

Ekspresija miR-346 u serumu se postepeno povećava tokom prelaska raka prostate u hormonsku rezistenciju, što ga čini potencijalnim biomarkerom za praćenje progresije raka prostate.

## Introduction

Prostate cancer (PCa) is one of the most common malignant tumours within the urinary system. Prostate biopsy is the gold standard for the diagnosis of PCa [1]. A biopsy is an invasive diagnostic procedure that can induce psychological distress in patients, including fear and discomfort [2]. Currently, the detection and screening of PCa biomarkers primarily include the following: (1) Detection of total prostate-specific antigen (tPSA) in serum; however, the results of tPSA are influenced by numerous clinical factors, leading to potential issues of overdiagnosis or missed diagnosis [3]; (2) Novel markers for PCa detection, such as RNA PCA3 and PHI, and the measurement of four kallikrein fractions have been preliminarily employed in PCa screening. Yet, they exhibit deficiencies in specificity and/or sensitivity [4] [5]. (3) Genetic tests, such as TMPRSS2-ERG, FOXA1, ZNF292, and CHD1, including the detection of DLX1 and HOXC6 mRNA expression levels in urine, have been initially applied in the clinical assessment of PCa. Still, they are not yet widely utilised [6] [7]. Additionally, the related problems in the progression of patients with PCa from hormone sensitivity to castration resistance have become research hotspots and difficulties, and the monitoring of related tumour markers has also become the focus of research. Therefore, it is necessary to identify more sensitive, specific, and non-invasive tumour markers to screen and monitor PCa progression.

MiRNAs are a class of highly conserved, non-coding small RNAs that play critical regulatory roles in gene expression and protein function in physiological and pathological processes [8]. Currently, the detection of miRNA markers is also used in clinical research on PCa. It can be used for the early diagnosis of PCa, risk assessment after endocrine therapy, and radical surgery. It can be combined with pathological morphology and immunohistochemistry to improve the prognosis stratification management plan [9] [10]. Furthermore, there is a related abnormal expression of microRNAs in the serum of patients with PCa [11]. The recent study by Fletcher Ce et al. [12] in which microRNA-346 (miR-346) was identified as an activator of androgen receptor (AR) signalling, which is directly associated with DNA damage response (DDR)-linked transcripts in PCa, suggests the critical potential of miR-346 in PCa. However, despite emerging evidence on miR-346 in other cancers [13] [14], its dual role in hormone-sensitive vs. castration-resistant PCa remains unexplored, particularly regarding its longitudinal expression during therapeutic transitions. Based on previous experiments [15] [16], this study explored its clinical significance and value as a serum marker of PCa by detecting the expression of microRNA-346 (miR-346) in tumour tissues and corresponding serum of patients with PCa, including the expression changes in the serum of patients with hormone-sensitive and castration-resistant PCa.

## Materials and methods

### Clinical materials

The sample size was calculated using G*Power (version 3.1) based on a detectable effect size of 0.3 (α = 0.05, β = 0.2), requiring 160 patients. Clinical data of 692 patients in Tengzhou Central People's Hospital from January 2017 to December 2023 were collected. The patients were grouped based on the inclusion criteria: (1) PCa group: the PCa was diagnosed by prostate biopsy, the clinical stage was T1-T3a, and the radical prostatectomy was conducted without any chemotherapy, radiotherapy, endocrine, surgical castration, and other related treatments before operation. (2) BPH group: patients with newly diagnosed benign prostatic hyperplasia underwent transurethral resection of the prostate, and postoperative pathological diagnosis was benign prostatic hyperplasia; (3) HSPC group: the patient was diagnosed with PCa by pathology, the clinical stage was T3b-T4, and no radical prostatectomy was performed. The PSA level decreased significantly in the first 6 months of regular endocrine therapy for 1 year, but there was no significant increase in the last 6 months. Additionally, whole-body bone imaging revealed that the patient's metastases had reduced. (4) CRPC group: In patients with PCa, after initial continuous endocrine therapy, serum testosterone reached the level of castration (<50 ng/dL), but the disease still progressed. (5) Control group: male healthy subjects of the same age who underwent physical examination in the physical examination centre of our hospital at the same time.

The exclusion criteria were as follows: with prostatitis or other acute infections; patients with abnormal blood pressure and blood glucose; complicated with severe cardiovascular and cerebrovascular diseases, pulmonary, liver, and kidney dysfunction; patients with severe bleeding tendency (platelet count <50x10^9^/L or INR >1.5) or blood coagulation disease; and complicated with mental illness or unable to cooperate with researchers.

A total of 48 patients who did not satisfy the conditions were excluded, and a total of 644 patients were included. The information on the study is shown in Table 1. Among them, the age of CRPC patients was higher than that of the other groups (*P*<0.05). Disease duration was significantly longer with disease progression: CRPC > HSPC > BPH > PCa (*P*<0.05). The tPSA of PCa with CRPC was higher than that of BPH, HSPC and controls (*P*<0.05). The PHI of PCa was higher than that of BPH and control (*P*<0.05). All patients involved in the study signed an informed consent form, and the study was approved by the Medical Ethics Committee of Tengzhou Central People's Hospital (No.: 2017-Ethical Review-18).

**Table 1:** Information on the study population. Note: a indicates *P*<0.05 compared with PCa patients, b indicates *P*<0.05 compared with BPH patients, c indicates *P*<0.05 compared with HSPC patients, and d indicates *P*<0.05 compared with CRPC patients.

Groups	n	Age	The course of<br>disease (moths)	tPSA (ng/mL)	PHI value
PCa	128	71.71±8.86	5.38±3.71	44.83±26.69	62.04±29.75
BPH	130	73.15±10.27	19.30±16.17^a^	4.15±3.70^a^	23.02±13.54^a^
HSPC	120	73.15±10.27	20.25±9.00^a^	3.54±2.45^a^	N/A
CRPC	116	78.24±6.80^abc^	36.48±15.02^abc^	45.43±22.60^bc^	N/A
Control	150	72.72±8.93^d^	N/A	1.02±0.87^abcd^	22.78±11.63^a^
F-value		9.241	135..524	286.519	174.524
*P*-value		<0.001	<0.001	<0.001	<0.001

### Main reagents and instruments

Trizol Kit: Invitrogen, TRI Reagent BD, purchased from Molecular Research Center, USA. Primers: U6snRNA (forward primer: 5'-CTCGCTTCGGCAGCACA-3', reverse primer: 5'-AACGCTTCACGAATTT-GCGT-3'), microRNA-346 (forward primer: 5' ACACTCCAGCTGGGTGTCTGCCCGCATGCC'3, reverse primer: 5' ACCAGGCTGGACAGTAGAG-CG'3). Reverse transcription reaction kit: PrimeScript RT reagent kit, real-time fluorescent quantitative PCR kit (SYBR Green PCR kit); all were purchased from Guangzhou Ruibo Biotechnology Co., Ltd. Real-time fluorescence quantitative PCR instrument: Applied Biosystems 7500, USA. PCR thermal cycler: Biometra. NanoDrop 2000 ultramicro spectrophotometer: nanodrop, USA.

### Specimen collection

Test specimens were collected for each group: preoperative serum and postoperative fresh PCa tissues of newly diagnosed patients with PCa; preoperative serum and postoperative fresh tissue samples of patients with BPH; serum of patients with HSPC at least 6 months after the start of endocrine therapy; serum of the patients on the day when he was diagnosed with CRPC; and serum of male healthy subjects of the same age who underwent physical examination in the physical examination centre of our hospital during the same period.

### Total RNA extraction

Total RNA was extracted from tissues and serum using the Trizol kit. The concentration and purity of RNA were determined via a Nanodrop 2000 ultramicro ultraviolet spectrophotometer. Then, the samples with the ratio of 260/A280 and A260/A230 in the range of 1.8-2.0 were selected for the next experiment. cDNA was synthesised via reverse transcription. The expression level of mir-346 in tissues and serum was detected via the real-time fluorescence quantitative method. Real-time fluorescence quantitative PCR results were obtained in the form of Ct values. All samples were amplified for microRNA-346 and U6 simultaneously; each sample was performed in three duplicate wells, and a blank reference was set. At the same time, single-hole NTC (no template control) detection was performed. Each experiment was repeated more than two-fold independently. The 2-^ΔΔCt^ method was used for analysis. RQ = 2^-ΔΔCt^, RQ represents the relative expression, ΔΔCt=ΔCt experimental group-Δ Ct reference group = (CtmiR-346-CtU6) experimental group- (CtmiR-346-CtU6) reference group.

### Prognostic follow-up

All patients were followed up for a minimum of 12 months of prognostic follow-up, which was done by regular review, with patients being asked to be reviewed at least once a month for the first 6 months and at least once every 2 months after 6 months.

### Observation indicators

The tPSA of each group: clinical stage, Gleason grade, and PHI of PCa; progress of PSA (serum PSA was monitored once every other week for three consecutive times. The PSA level continued to rise and increased by more than 50% compared to the baseline value. Concurrently, the absolute value of PSA reached more than 2 ng/mL); the progress of new lesions on imaging examination and ALP in the HSPC and CRPC groups were compared. Hormone resistance conversion time (refers to the time of the whole process of hormone-sensitive patients with PCa receiving standardised endocrine therapy, through disease progression, and finally being diagnosed as castration-resistant PCa) and life expectancy in the CRPC group were also compared.

### Statistical analysis

GraphPad Prism statistical analysis software was used to analyse the experimental data, and the relative expression of miR-346 was expressed by (x̄±s). The independent sample t-test was used to compare the two groups, and a one-way analysis of variance was used to compare multiple groups. Spearman bivariate rank correlation analysis was used to analyse the correlation between the expression of miR-346 in tissues and corresponding serum and the correlation between the expression of miR-346 and tPSA, PHI, clinical stage, Gleason grade, ALP conversion time, and life expectancy. Post-hoc comparisons were performed using Tukey's honest significant difference test to adjust for multiple comparisons. Statistical significance was set at a value of *P*<0.05.

## Results

### Expression of miR-346 in tissues and corresponding serum of the PCa group and BPH group

**Figure 1:**
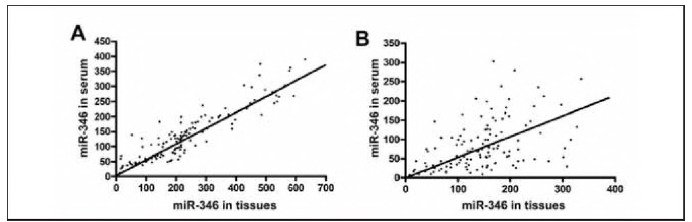
Correlation analysis of the expression of miR-346 in BPH, PCa tissues, and corresponding serum.<br>A: The expression of miR-346 in BPH tissues was positively correlated with that in the corresponding serum; B: The expression of miR-346 in PCa tissues and corresponding serum was also positively correlated

The expression of miR-346 in PCa tissues was significantly lower than that in BPH tissues, but there was no significant difference in the expression of serum miR-346 between the BPH and control group (t=0.65, *P*=0.52); however, they were significantly higher than those in the PCa group (t* = 8.19, P*<0.001; t^#^=5.98, *P^#^* <0.001) (Table 1). The expression of miR-346 in the tissues of patients with BPH was positively correlated with that in the corresponding serum (Figure 1A, Table 2). Similarly, miR-346 expression in cancer tissues of newly diagnosed patients with PCa was also positively correlated with that in the corresponding serum (Figure 1B, Table 1).

**Table 2:** The difference and correlation of miR-346 expression in tissues and serum of PCa, BPH, and the control group. Note: * indicates P<0.05 compared with the control group, and # indicates P<0.05 compared with BPH group.

Groups	Relative expression<br>of miR-346 in tissues	Relative expression of <br>miR-346 in serum	t-value	*P*-value
PCa	144.80±72.85	82.61±62.82*#	0.5060	<0.001
BPH	237.60±146.00	137.40±82.85	0.8914	<0.001
Control	N/A	132.50±37.31	N/A	N/A
t or F -value	6.443	30.61		
p-value	<0.001	<0.001		

### Correlation analysis between the expression of miR-346 and tPSA, PHI, clinical stage, and Gleason grade in PCa tissues and corresponding serum

**Figure 2:**
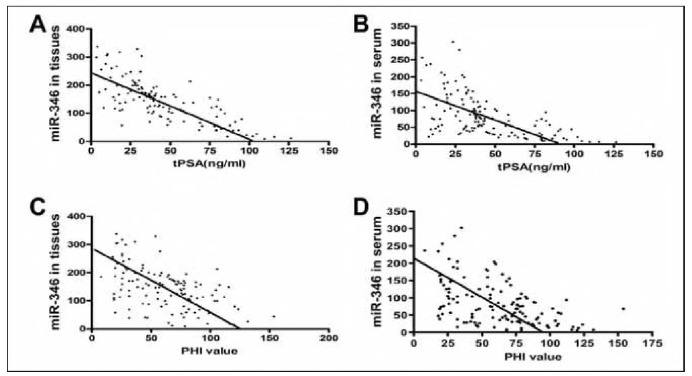
Correlation analysis between the expression of miR-346 and tPSA, PHI.<br>A and B: The expression of miR-346 in PCa tissues and serum was negatively correlated with tPSA; C and D: The expression of miR-346 in PCa tissues and serum was negatively correlated with the PHI value.

The expression of miR-346 in PCa tissues and serum was not correlated with age and course of disease but negatively correlated with tPSA (r_tissue_=0.7486, r_serum_ = 0.6213, *P*<0.001) (Figure 2A, Figure 2B) and also negatively correlated with PHI (r_tissue_=0.5034, r_serum_=0.4928, *P*<0.001) (Figure 2C, Figure 2D). The expression of miR-346 in PCa tissues and serum was significantly different between different clinical stages and Gleason grades (Table 3). Moreover, the expression of miR-346 in PCa tissues and serum was negatively correlated with the clinical stage (r_tissue_ = 0.3458, *P*<0.001; r_serum_ = -0.3051, P = 0.0005). and Gleason grade (r_tissue_=0.6940, *P*<0.001; r_serum_=0.3831, *P*<0.001).

**Table 3:** The relationship between the expression of miR-346 in PCa tissues/serum and clinical stage and Gleason grade. Note: a indicates *P*<0.05 compared with Phase I, b indicates *P*<0.05 compared with Phase II, c indicates *P*<0.05 compared with Phase III; d indicates *P*<0.05 compared with Gleason 1, e indicates *P*<0.05 compared with Gleason 2, f indicates *P*<0.05 compared with Gleason 3, and g indicates *P*<0.05 compared with Gleason 4.

Category	number of<br>samples	Relative expression<br>of miR-346 in tissues	F-value	*P*-value	Relative expression<br>of miR-346 in serum	F-value	*P*-value
Clinical stages							
Phase I	33	186.00±80.23	18.89	0.0003	116.60±79.39	10.60	0.0141
Phase II	39	141.30±65.27^a^			78.67±50.51^a^		
Phase	32	142.70±56.74^a^			69.94±53.43^ab^		
Phase	24	96.71±63.95^abc^			61.75±47.94^abc^		
Gleason grade							
1	33	216.30±88.86	61.05	<0.0001	126.10±88.24	21.52	0.0003
2	37	193.50±49.73^d^			104.60±58.98^d^		
3	36	135.60±47.51^de^			85.47±73.12^de^		
4	27	91.30±45.69^def^			52.61±35.03^def^		
5	14	73.50±53.77^defg^			49.36±36.54^defg^		

### The expression and clinical significance of serum miR-346 in patients with PCa, HSPC, and CRPC

**Figure 3:**
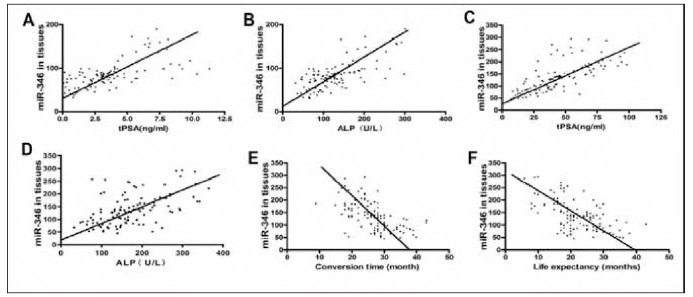
The expression and clinical significance of serum miR-346 in patients with PCa, HSPC, and CRPC.<br>A and B: The correlation between tPSA, ALP and the expression of miR-346 in serum of patients with HSPC. C and D: The correlation between tPSA, ALP and the expression of miR-346 in serum of patients with CRPC. E and F: The correlation between the conversion time, life expectancy, and the expression of serum miR-346 in patients with CRPC.

The relative expression levels of serum miR-346 in patients with PCa, HSPC, and CRPC were (82.61 ±62.82), (83.00±30.44), and (137.00±59.10), respectively. There was no significant difference in the expression of serum miR-346 between the PCa group and the HSPC group (t=0.06, P=0.95); however, they were significantly lower than the CRPC group (tPCa = 6.95, PPCa < 0.001; tHSPC = 8.87, PHSPC<0.001). Serum miR-346 expression in patients with PCa was negatively correlated with tPSA levels (previously described). However, the expression of serum miR-346 in HSPC patients was positively correlated with tPSA (3.54±2.45 ng/mL) (r=0.6839, P<0.001) (Figure 3A) and ALP (128.40±60.69U/L) (r=0.6183, P<0.001) (Figure 3B). Similarly, the expression of serum miR-346 in patients with CRPC was positively correlated with tPSA (45.43±22.60 ng/mL) (r=0.7899, P<0.001) (Figure 3C) and ALP (172.972.84 U/L) (r=0.5411, P<0.001) (Figure 3D).

The conversion time from hormone sensitivity to castration resistance in 116 patients with CRPC was (26.46±6.35) months, with a median time of (27.00) months, which was negatively correlated with the expression of serum miR-346 in patients with CRPC (r=-0.6575, *P*<0.001) (Figure 3E). The life expectancy of patients with CRPC was (22.04±7.11) months, and the median time was (21.00) months, which was also negatively correlated with the expression of serum miR-346 in patients with CRPC (r=-0.5699, *P*<0.001) (Figure 3F). The higher the relative expression of serum miR-346 detected when the patient was diagnosed with CRPC, the shorter the duration of the patient's transition from hormone sensitivity to castration resistance and the shorter the life expectancy.

## Discussion

This study identifies miR-346 as a dynamic biomarker in PCa progression, with lower expression in primary tumours (vs. BPH) but higher expression in CRPC (vs. HSPC). These findings suggest a dual role of miR-346 in hormone-sensitive vs. resistant stages.

Serum miRNAs are expected to replace miRNAs in tumour tissues and can be used for the diagnosis, monitoring, and elucidation of biological molecular mechanisms of PCa. Meanwhile, as serum has the characteristics of convenient sampling, minimal trauma, and repeated detection, serum miRNA detection has the potential advantages of simplicity, safety, ease of use, and accuracy. Therefore, serum miRNA detection has greater clinical research advantages than tissue miRNA detection.

miR-346 has been demonstrated to play an essential role in various diseases. For example, Wang et al. [17] found that miR-346 regulates the osteogenic differentiation of human bone marrow-derived mesenchymal stem cells by regulating GSK-3β and activating the Wnt/β-catenin pathway. Wolter et al. [18] detected that the expression levels of target genes TERT and SEMA6A regulated by miR-346 were significantly increased by inhibiting the expression of miR-346 in brain malignant glioma cells, which promoted the invasion and metastasis of brain malignant glioma cells. Similarly, our study also found that the expression of miR-346 in tissues and corresponding serum of patients with PCa was significantly lower than that in patients with BPH and was negatively correlated with PSA, PHI value, clinical stage, and Gleason grade, indicating that miR-346 may play a »tumour suppressor gene« role in PCa pathogenesis. Although our findings suggest a strong correlation between miR-346 and clinical parameters, causality remains unproven. Future functional studies (e.g., CRISPR-Cas9-mediated miR-346 knockdown) are needed to elucidate its mechanistic role.

Moreover, in some tumours, miR-346 also plays the role of »oncogene.« In the study of cervical cancer, Guo et al. [19] found that miR-346 can not only promote AGO2 gene expression but also enhance the role of other microRNAs and cooperate with them to improve the invasion ability of cervical cancer cells and promote the progression of cervical cancer. A study by Sun JC et al. [20] demonstrated that miR-346 can regulate the proliferation, migration and invasion of non-small cell lung cancer cells. The study by Zhang X et al. [21] found that circ_0026628 acted as an endogenous sponge for miR-346 and FUS to enhance SP1 expression at the post-transcriptional level, which ultimately promoted colorectal cancer development. Similarly, our previous study found that miR-346 was remarkably expressed in castration-resistant PCa cell lines than in hormone-dependent PCa cell lines [15] [16].

Additionally, clinical studies have found that serum miR-346 expression is significantly higher in patients with CRPC than in patients with PCa and HSPC, indicating that it plays an oncogenic role in PCa progression. The elevated miR-346 in CRPC may reflect adaptive responses to endocrine therapy pressure rather than mere disease progression. For instance, androgen deprivation upregulates miR-346 through RIP140/NF-B signalling [22], which in turn promotes neuroendocrine differentiation.

Whether miR-346 acts as a tumour suppressor or an »oncogene« in PCa depends on its involvement in different cellular signal transduction pathways and molecular biological mechanisms. The TGFβ signalling pathway is closely related to the occurrence and development of PCa. TGFβ signalling pathway affects the progression of epithelial-mesenchymal transition (EMT) and whether TGFβ acts as a tumour promoter or suppressor in PCa progression [23]. According to TargetScan records, miR-346 has a high correlation coefficient with SMAD3 and SMAD4 genes in the TGFβ signalling pathway, and SMAD3 and SMAD4 genes are closely related to the EMT process [24]. It can be speculated that the occurrence and progression of PCa are also related to the regulation of SMAD3 and SMAD4 genes by miR-346.

Additionally, TGFβ plays a dual role in the occurrence and development of tumours. In some tumours, it plays a »carcinogenic role« by promoting tumour progression and metastasis by promoting tumour vascular epithelial cell formation and inhibiting the immune response. However, in other tumours, it acts as a tumour suppressor by inhibiting epithelial cell growth or plays a different role at different stages of progression of the same tumour [25] [26] [27]. Similarly, the combination of miR-346 and different genes in PCa plays different roles in carcinogenesis or as a »tumour suppressor«. Studies have confirmed that miR-346 can upregulate RIP140 protein activity by targeting the 5'-untranslated region of RIP140 protein mRNA [28]. RIP140 activation improves the activity of the ligand-binding region and inhibits androgen dependence on androgen receptors, which in turn promotes the transformation of castration resistance in PCa [22]. Our study also found that miR-346 acts as a tumour suppressor in patients with PCa before receiving endocrine therapy. However, miR-346 plays the role of »oncogene« in the conversion of hormone resistance in patients with PCa after a period of endocrine therapy. Hence, we speculated that miR-346 plays a dual role as a tumour suppressor and an oncogene in the occurrence and progression of PCa.

However, the study warrants further improvement. The selected subjects included patients with PCa with different stages of the disease, and we failed to systematically collect data for each patient from the initial diagnosis of PCa to HSPC until the CRPC stage and failed to detect and analyse the expression of serum miR-346 during the entire process of progression of the same patients with PCa. Additionally, our study only found an apparent relationship between the expression of serum miR-346 and clinical parameters, such as tPSA and ALP, in patients with PCa; however, the causal relationship was unclear. This study still needs to conduct further research on the cell signal transduction pathway and molecular biological mechanism of miR-346 in the pathogenesis and progression of PCa.

## Conclusion

miR-346 can both be a biomarker for early diagnosis of PCa and be used to monitor the process of hormone sensitivity resistance in PCa, providing a new monitoring method for the progression and prognosis of this disease. However, further validation of its diagnostic efficacy compared to established markers (e.g., PCA3) is needed.

## Dodatak

### Acknowledgements

Not applicable.

### Availability of data and materials

Not applicable.

### Funding

This study was supported by the National Natural Science Foundation of China (82171594) Shandong Province Medicine and Health Science and Technology Development Project (grant number 202204050416), and The Affiliated Hospital of Xuzhou Medical University Development Fund Project (grant number XYFY2020046).

### Authors' contributions

Conception and design of the research: Xiaoqiang Liu; Acquisition of data: Lei Wang, Yanqing Wang, Li Tian; Analysis and interpretation of data: Lei Wang, Zhengwei Wang, Li Tian; Statistical analysis: Lei Wang, Li Tian, Guangzhou Cheng; Revision of manuscript for important intellectual content: Xiaoqiang Liu; Drafting the manuscript: Lei Wang.

### Ethics approval and consent to participate

The study was approved by the Medical Ethics Committee of Tengzhou Central People's Hospital (Ethical No.: 2017-Ethical Review-18).

### Patient consent for publication

All patients involved in the study signed an informed consent form and agreed to publish the paper.

### Conflict of interest statement

All the authors declare that they have no conflict of interest in this work.
